# High-throughput computation of Raman spectra from first principles

**DOI:** 10.1038/s41597-023-01988-5

**Published:** 2023-02-08

**Authors:** Mohammad Bagheri, Hannu-Pekka Komsa

**Affiliations:** Microelectronics Research Unit, Faculty of Information Technology and Electrical Engineering, University of Oulu, Oulu, FIN-90014 Finland

**Keywords:** Condensed-matter physics, Theory and computation, Raman spectroscopy

## Abstract

Raman spectroscopy is a widely-used non-destructive material characterization method, which provides information about the vibrational modes of the material and therefore of its atomic structure and chemical composition. Interpretation of the spectra requires comparison to known references and to this end, experimental databases of spectra have been collected. Reference Raman spectra could also be simulated using atomistic first-principles methods but these are computationally demanding and thus the existing databases of computational Raman spectra are fairly small. In this work, we developed an optimized workflow to calculate the Raman spectra efficiently and taking full advantage of the phonon properties found in existing material databases. The workflow was benchmarked and validated by comparison to experiments and previous computational methods for select technologically relevant material systems. Using the workflow, we performed high-throughput calculations for a large set of materials (5099) belonging to many different material classes, and collected the results to a database. Finally, the contents of database are analyzed and the calculated spectra are shown to agree well with the experimental ones.

## Background & Summary

Raman spectroscopy is a widely used, powerful, and nondestructive tool for analysis and identification of materials as well as assessing material quality. It is based on characterization of the vibrational modes of materials and provides rich atom- or chemical bond-specific information about the crystal structure and chemical composition. When used in assessing material quality, Raman spectra contains information about grain sizes, defect densities, and strain, among others^1–4^. In other fields, Raman spectroscopy has been used to, e.g., detect counterfeit medicines, identify plastic types in recycling flows, to detect hazardous chemicals, or to measure temperature^5–9^. Raman spectrum provides a fingerprint of the material, but it is usually not possible to directly interpret e.g. the material composition from the spectrum. In order to use Raman in the above-mentioned material classification and identification applications, a database of known reference spectra is needed. To this end, databases of experimental spectra have been collected, such as the RRUFF Project^10^ that contains a large set of experimental Raman spectra of minerals (4112 public samples), KnowItAll Raman Spectral Library^11^ that include Raman spectra of different organic and inorganic compounds, polymers and monomers (over 25000 records), and Raman Open Database(ROD)^12^ which complements the crystallographic information found in the Crystallographic Open Database (COD)^13^ (1133 entries).

A Raman Spectrum database made via ab initio, density-functional theory (DFT) electronic structure calculations could be highly useful in providing supplementary information that is difficult to obtain from experiments. For instance, some materials can be difficult to synthesize in a pure form, or their purity or phase content is unknown. The calculated results are also free of any instrumental contributions. Computational studies can also be faster and cheaper to carry out than experiments. Such a database would also be useful to computational researchers, e.g., by providing a reference spectra. Moreover, large datasets can be used in material informatics for material screening or for training models via machine-learning. Still, compared to the experimental ones, the computational databases are of very limited size. This is due to the computational cost of these calculations, which makes them limited to small systems and/or a small number of materials. A few open-access libraries of computational Raman spectra already exist such as: (i) Computational 2D Materials Database (C2DB)^9,14^ that contains properties of a large number of 2D materials but only 733 structures have Raman spectra, (ii) WURM project^15^ is a database of computed Raman and infrared spectra for 461 minerals, and (iii) in developing high-throughput computational methods, Liang *et al*. calculated 55 inorganic compounds^16^.

In this paper, we report on our research to develop optimized high-throughput workflow to carry out the Raman tensor calculations and build a large database of computational Raman spectra, while taking advantage of the calculated phonon properties in Phonon database^17^. For selected systems, the calculated spectra are compared to those obtained using previous computational methods as well as to the experimental ones reported in the literature. The database of Raman spectra and vibrational properties reported along with this paper consists of 5099 compounds from many different material classes, far surpassing in size the previous computational databases and comparable to the experimental ones.

## Methods

### Simulation of Raman spectra

In Raman spectroscopy measurements, incident laser photons with a specific frequency *ω*_*L*_ interact with lattice vibrations, described in the form of phonons in crystalline materials, and the spectrum of inelastically scattered photons are recorded. Scattered photons exhibit either a decrease in frequency *ω*_*S*_ upon creation of phonon or increase in frequency upon annihilation of a phonon, denoted as Stokes or Anti-Stokes shifts, respectively. The intensity of the peaks is related to the Raman scattering cross section, which can be challenging to calculate since the ion (and electron) dynamics in the material need to be described concurrently with the light-matter interaction^18,19^.

There are several approaches for calculating the Raman spectra: (i) scattering probability from third-order perturbation theory (absorption, electron-phonon coupling, and emission)^9,20,21^, (ii) from the gradient of the electronic susceptibility (usually via finite-differences) in Placzek approximation^21–23^, and (iii) from the auto-correlation function of time-dependent susceptiblity^24,25^. Methods (i) and (ii) only yield the Raman tensor, but the phonon eigenvectors and frequencies need to be determined first in a separate calculation step. In method (iii), the peak positions and intensities are obtained at once, but it is computationally highly demanding. Method (ii) is computationally most affordable and easy to implement in high-throughput setting^16^ and thus adopted in this work. The method is briefly described below.

In the first step, the phonons are calculated as described in depth in many previous publications^26,27^. Within harmonic approximation, the potential energy surface is written as a Taylor expansion $U={U}_{0}+{\Phi }_{\alpha \beta }(ki,lj){u}_{\alpha }(ki){u}_{\beta }(lj)$, where *U*_0_ is the ground state energy and force constant matrix Φ describes the second-order change in potential energy,

1 $${\Phi }_{\alpha \beta }\left(ki,lj\right)=\frac{{\partial }^{2}U}{\partial {u}_{\alpha }(ki)\partial {u}_{\beta }(lj)}=\frac{\partial {F}_{\alpha }(ki)}{\partial {u}_{\beta }(lj)}$$

In Eq. (1), *u*_*α*_*(ki)* is the displacement of the *k* th atom in the *i* th unit cell in the cartesian direction *α*. *F*_*α*_(*ki*) is the force in atom *ki*, and in the equation above its change is induced by the displacement of atom *lj*. After harmonic ansatz for the temporal evolution of the vibrational modes *v*, the classical equations of motion for atoms in unit cell “0” become

2 $${M}_{k}{\omega }^{2}{v}_{\alpha }(k0)=\sum _{l,j,\beta }{\Phi }_{\alpha ,\beta }(k0,lj){v}_{\beta }(lj)$$

where *M*_*k*_ is the mass of atom *k*. The infinite sums over unit cells *l* in periodic crystals can be avoided by moving to reciprocal space and, after rescaling *v* and Φ by $\sqrt{M}$, Eq. 2 is cast into an eigenvalue equation

3 $$\sum _{l\beta }{D}_{\alpha \beta }\left(kl,q\right){e}_{\beta }(l,q\nu )={[\omega (q\nu )]}^{2}{e}_{\alpha }(k,q\nu )$$

where *D* is the mass-scaled Fourier-transformed Φ (denoted dynamical matrix), *q* is the wave vector, *e* is the eigenvector of the band index *ν*, and *ω*^2^ are the eigenvalues. To obtain *D*, force constants Φ need to be evaluated from the forces induced at atoms *lj* by displacing each atom *k*0 in the unit cell. To guarantee sufficiently large distance between atoms *k*0 and *lj*, supercell calculations are usually required. If the crystal symmetry is not considered, the construction of the force constant matrix requires performing 3*N* DFT calculations when each of the *N* atoms in the unit cell is displaced in each of the three cartesian directions.

Differential cross section for the Stokes component of Raman scattering from the *ν* th eigenmode far from resonance is given as^18,23^

4 $$\frac{d{\sigma }_{\nu }}{d\Omega }=\frac{{\omega }_{S}^{4}{V}^{2}}{{(4\pi )}^{2}{c}^{4}}{\left|{\widehat{E}}_{S}\frac{\partial \chi }{\partial {\xi }_{\nu }}{\widehat{E}}_{L}\right|}^{2}\frac{\hbar (n+1)}{2{\omega }_{\nu }}$$

where ${\widehat{E}}_{S}$ and ${\widehat{E}}_{L}$ are the unit vectors of the polarization for the scattered and the incident light, V is scattering volume, *n* is the Bose-Einstein statistical factor, *ξ* is a normal-mode coordinate along the mass-scaled eigenvector ${e}_{\alpha }^{{\prime} }(k)={e}_{\alpha }(k)/\sqrt{{M}_{k}} \sim {v}_{\alpha }(k)$ and *χ* is the electronic susceptibility tensor. The directional derivative can be written out as

5 $$\begin{array}{l}\frac{\partial \chi }{\partial \xi }=\nabla \chi \cdot e{\prime} =\mathop{\sum }\limits_{k}^{unitcell}\frac{\partial \chi }{\partial {u}_{\alpha }(k)}{M}_{k}^{-\frac{1}{2}}{e}_{\alpha }(k)\approx \frac{\chi ({R}_{0}+h{\prime} e{\prime} )-\chi ({R}_{0}-h{\prime} e{\prime} )}{2h{\prime} }\\ \,\,\,=\frac{\chi ({R}_{0}+h\widehat{e{\prime} })-\chi ({R}_{0}-h\widehat{e{\prime} })}{2h}| e{\prime} | \end{array}$$

The first two forms involve calculation of derivatives of *χ* with respect to displacement of each atom *u*(*k*), whereas in the last two forms all atoms are displaced simultaneously along *e*′ and explicitly written in the finite-difference approximation as implemented in the code (displacing the atoms in both positive and negative directions). Normalized $\widehat{e{\prime} }=e{\prime} /| e{\prime} |$ (and $h=h{\prime} | e{\prime} |$) is used in order to have consistent step size *h* in systems and modes with different masses (and in units of Å).

Specifically, the Raman tensor is defined as^23^

6 $${R}_{\nu \beta \gamma }=\frac{{V}_{c}}{4\pi }\frac{\partial {\chi }_{\beta \gamma }}{\partial {\xi }_{\nu }}$$

incorporating *V*^2^/(4π)^2^ from Eq. (4). To evaluate the change in *χ*, we used the macroscopic dielectric constant ${\varepsilon }_{\beta \gamma }$ containing only the electronic contribution with clamped ions (sometimes denoted as the high-frequency dielectric constant *ε*_∞_), which is readily provided by most DFT codes.

While the expression in Eq. (4) yields complete information, quite often experimental results are obtained for polycrystalline mineral specimens or powdered samples, in which case the intensity must be averaged over all possible orientations of the crystals. We adopt a commonly used measurement configuration where the direction of incident light, its polarization, and the direction of outgoing light are all perpendicular, and the Raman intensity can then be written as^21,23^

7 $$\frac{d{\sigma }_{\nu }}{d\Omega }=\frac{{\omega }_{S}^{4}}{{c}^{4}}\frac{\hbar (n+1)}{2{\omega }_{\nu }}\frac{{I}_{{\rm{R}}aman}}{45}$$

where

8 $${I}_{{\rm{R}}{\rm{a}}{\rm{m}}{\rm{a}}{\rm{n}}}=45{a}^{2}+7{\gamma }^{2}$$

9 $$a=\frac{1}{3}\left({R}_{\nu xx}+{R}_{\nu yy}+{R}_{\nu zz}\right)$$

10 $${\gamma }^{2}=\frac{1}{2}\left[{\left({R}_{\nu xx}-{R}_{\nu yy}\right)}^{2}+{\left({R}_{\nu xx}-{R}_{\nu zz}\right)}^{2}+{\left({R}_{\nu yy}-{R}_{\nu zz}\right)}^{2}+6\left({R}_{\nu xy}^{2}+{R}_{\nu xz}^{2}+{R}_{\nu yz}^{2}\right)\right]$$

*I*_*Raman*_ is Raman activity that is independent of experimental factors such as temperature and incoming photon energy and thus used when comparing our results to other calculations, whereas Eq. 7 is used (and must be used) when comparing to experimental spectra. We plot the spectra at 300 K and assuming *ω*_*L*_ > *ω*_*ν*_, in which case the *ω*_*S*_ term becomes nearly constant and vanishes after normalization.

### Workflow

We now describe how the theory described above is turned to an efficient computational workflow. As mentioned, the computational procedure involves two sets of calculations: (i) force constants to get the vibrational modes and (ii) the Raman tensors for each mode. While the phonons at Γ-point can be calculated efficiently, we would like to have access to the full force constant matrix. This allows calculation of phonon dispersion and also, e.g., estimation of isotope effects and line broadening due to defects or grains via phonon confinement model^18,28–30^. Both steps can be computationally demanding for systems with large number of atoms in the unit cell, which has hindered previous efforts to building such databases in the past.

The most important design decisions that distinguish our work from the previous ones are the following. First, we have decided to build our database on top of the Atsushi Togo’s Phonon database^17,31^, that contains the calculated full force constant matrix, and our work only focuses on calculating the Raman tensors. We are using the same computational parameters, and thus our database is fully consistent with the Phonon database, which is further linked to the Materials project database^32^ via the material-IDs.

Second, to reduce calculation time and make the workflow more efficient compared to existing methods, Raman-active modes are found based on group theory and the Raman tensors are calculated only for modes that are known to be active or whose activity could not be determined. Known inactive modes and the three zero-frequency acoustic modes are ignored. For this purpose, the symmetry information about Raman activity was implemented. A mode is Raman-active if its irreducible representation (irrep.) basis functions are quadratic (*xy*, *x*^2^ etc.). We extracted this information from the point group tables of the Bilbao Crystallographic Server^33^ and they are listed in Table S2. When the irrep. of the mode could not be determined, consequently also the Raman activity was marked unknown.

**Fig. 1:**
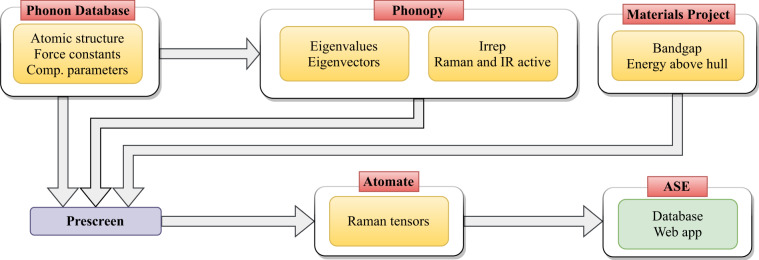
High-throughput calculation workflow, grouped according to the software or database used (red box) and the light yellow boxes indicating the relevant material properties.

The workflow developed for automatic Raman tensors calculations is illustrated in Fig. 1. At the conceptual level, the workflow steps are following:

1. Select material from Phonon database, read in optimized structure, computational parameters, and force constant matrix.
2. Calculate the eigenvectors and eigenvalues at Γ-point.
3. Determine the irreducible representation of the modes and whether they are Raman and/or infrared active.
4. Perform prescreening to check that the material is dynamically and thermodynamically stable and the material is not metallic or near-metallic.
5. Calculate the Raman tensors for Raman-active modes and the dielectric tensors for the optimized structure.
6. All the results (structure, eigenvalues, irreducible representation, Raman tensors, etc.) are collected in a database.

The softwares used in each step are also indicated in Fig. 1. Atsushi Togo’s Phonon database contains the optimized structures, calculated force constants, and all the computational parameters used to obtain them. These are calculated using VASP software^34,35^. The eigenvalues and eigenvectors at Γ-point, as well as the irreducible representations of the modes are calculated using Phonopy^31^. All of this information together with selected material properties obtained from the Materials Project database are collected in a database for prescreening. For this, we adopted to use the database tools in atomic simulation environment (ASE)^36^. In the last step, the calculated Raman tensors are added to this database, which is then also served through a web app implemented in ASE and developed as a part of Atomic Simulation Recipes (ASR)^37^.

For automating the computationally intensive part, i.e., the calculation of the Raman tensors, we used the Atomate^38^ that is a Python-based package for constructing complex materials science computational workflows. The workflow objects generated by Atomate are given to Fireworks workflow software^39^ for managing, storing, and executing them with the help of Custodian package for error management^40^. As the DFT calculator we used here VASP, with the parameters taken from the Phonon database. During these calculations, all the input parameters and results are stored in a Mongo database, which are afterwards transferred to the database (Computational Raman Database, CRD).

### Prescreening

**Fig. 2:**
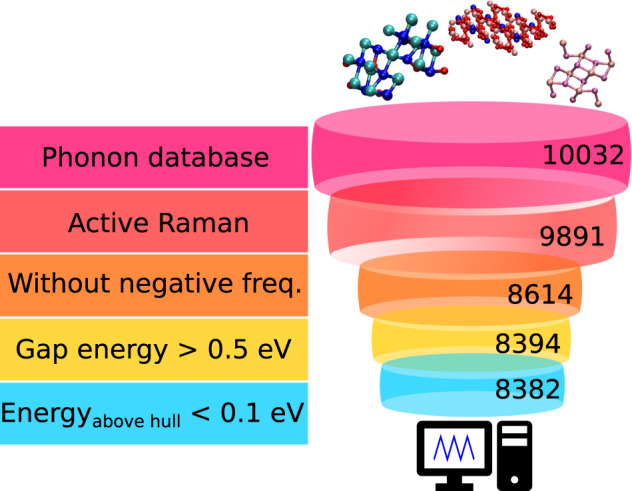
Structure selection procedure. The prescreening criteria are indicated on the left and the the number of structures in each step are indicated on the right.

Before Raman tensor calculations we performed the following prescreening, also illustrated in Fig. 2: (i) We check that the material has Raman active mode(s) based on the symmetry analysis. (ii) We check that the material is dynamically stable, i.e., there are no modes with imaginary frequencies at the Γ-point. (iii) We check that the material is thermodynamically stable by requiring that the energy above the convex hull is less than 0.1 eV/atom, as materials with the energy >0.1 eV are unlikely to be experimentally synthesized^41^. (iv) We check that the bandgap is larger than 0.5 eV, since our computational approach is strictly valid only for non-resonant conditions (i.e., photon energy smaller than the band gap), and metallic systems require very large k-point meshes which will increase the computational cost. For (iii) and (iv) we use information from the Materials Project database at the same material ID^32^. Finally, we have 8382 (83.55%) materials satisfying these conditions and flagged for calculation. It is also worth noting that Phonon database contains only materials that are non-metallic, non-magnetic, and non-triclinic.

The workflow first performs calculation of dielectric tensors of the optimized structure, which can be compared to that provided in Phonon database. Additionally, the maximum forces are checked in this step and the calculation terminated if the forces are >0.001 eV/Å, but no such case was encountered.

### Computational parameters

All density-functional theory (DFT) calculations are carried out using VASP (Vienna Ab initio Simulation Package)^34,42^ with projector-augmented wave method^43^. PBEsol exchange-correlation functional^44^ and other computational parameters were taken to be the same as used in Phonon database. In particular, plane wave cutoff is set to 1.3 times the maximum cutoff listed in PAW setups. In Phonon database, the structures of standardized unit cells are given, whereas we adopt to use the primitive cell in Raman tensor calculations to save computational time. The primitive cell can be readily obtained using Phonopy^31^. In the calculation of eigenvectors, non-analytic corrections are not included, as the eigenvectors would then depend also on the direction from which *q* → 0 is approached and thereby complicate the calculations significantly. Fortunately, this mostly happens for the IR-active modes and less for the Raman-active modes. Moreover, the induced change in eigenvectors and in Raman tensors is expected to be small and the splitting of the modes can be determined a posteriori.

There are then only two parameters left to decide: the k-point mesh and the magnitude of the atomic displacements in evaluation of the Raman tensor by finite differences.

In Phonon database, the Brillouin zone of the unit cell is sampled by a Γ-centered k-point mesh whose density is defined by the *R*_*k*_ parameter in VASP as a length that determines the subdivisions *N*_1_, *N*_2_, and *N*_3_ along the reciprocal lattice vectors *b*_1_, *b*_2_, and *b*_3_, respectively, via ${N}_{i}={\rm{\max }}(1,{R}_{k}| {b}_{i}| +0.5)$ and rounded to an integer. We adopt the same approach, but it is worth noting that since we use primitive cell, the exact density and positions of mesh points can be slightly different. Moreover, metals and small-gap semiconductors usually require higher density k-point mesh than large-gap insulators. All calculations in the Phonon database used *R*_*k*_ = 20, which should be sufficient for the structural optimization of materials included in the database (band gap > 0.5 eV). Determination of Raman tensor may, however, require a higher value. In order to benchmark this, we selected two materials from the Phonon database with different band gaps: the largest band gap material among the common III-V semiconductors is AlN (4.05 eV) and Si is a small band gap material (0.85 eV).

As illustrated in Fig. S1, *R*_*k*_ = 40 is needed to achieve converged results for dielectric constant and Raman intensity of a small band gap material Si, whereas for a large band gap material AlN *R*_*k*_ = 20 is sufficient. See Benchmark section in SI for more details. In our workflow, we have chosen to use the following values: *R*_*k*_ = 20 for the structures with a band gap more than the 2 eV, *R*_*k*_ = 30 for band gaps in the range of 1–2 eV, and *R*_*k*_ = 40 for band gaps smaller than 1 eV.

In order to benchmark the displacement, we chose materials with heavy and light elements, PbO and Cd(HO)_2_. As shown in Fig. S2, varying the displacement from 0.001 Å to 0.04 Å (default value being 0.005 Å), we found little change in the Raman tensors or the dielectric constants. Therefore, we chose to use the default value. Finally, we verified the computational workflow in Atomate by comparing the Raman spectra of few structures to those obtained using VASP_Raman code^45^. As shown in Fig. S3, a good agreement is found. We note that Atomate had wrong normalization of eigenvectors which in some cases resulted in overestimation of the Raman intensities, but was fixed in the version used here.

## Data Records

### Computational Raman database

The final database contains vibrational information and Raman tensors stored in JSON format, a simple and lightweight text-based data format, which can be downloaded directly from the Materials Cloud Archive^46^. The Table 1 shows all the database keys with their related descriptions which could be used for navigating as nested key/value pair with a simple python script. The JSON document also includes the structure data (along with point and space group symmetries) and additional properties derived from our calculations (Raman/IR activity list, bandgap, dielectric constants, and dimensionality), from Phonon database (eigenvectors, Γ-point frequencies, and Born charges) and from Materials Project database (bandgap, dielectric constants, and energy above convex hull). Links to the relevant entries in these external databases are also included.

**Table 1:** Description of the JSON file structure for Computational Raman Database.

Keys	Datatype	Description
lattice_parameters	list	*a*, *b*, and *c* lattice constants (Å)
lattice_angles	list	*α*, *β* and *γ* angles between lattice vectors
cell	array	Lattice vectors in 3 × 3 matrix format
positions	array	Atomic positions in relative coordinates
numbers	array	Total number of atoms and atomic numbers of all elements
mass	float	Sum of atomic masses in the unit cell (amu)
volume	float	Volume of the unit cell (Å^3^)
mpid	string	MP ID
bandgap_mp	float	Band gap from MP database (eV)
bandgap	float	Band gap (eV)
cbm	float	Conduction band minimum (eV)
vbm	float	Valence band maximum (eV)
diel_mp	array	Dielectric tensor (electronic contribution) from MP database
diel	array	Dielectric tensor (electronic contribution)
frequencies_thz	list	Γ-point frequencies (THz)
frequencies_cm	list	Γ-point frequencies (1/cm)
pointgroup	string	Point group
spacegroup	string	Space group
chemical_formula	string	Chemical formula
IRactive	array	Infrared-active modes
IRlabels	list	Irreducible representation (irrep.) labels of modes
IRbands	list	Irrep. band groups of degenerate modes
natom	integer	Total number of atoms
Ramanactive	array	Raman activity of modes (0: inactive, 1: active, −1:unknown)
raman_tensors	array	Raman tensors
born	array	Born charges (*e*)
eigenvec	array	Eigenvectors
dimensionality	string	Dimensionality of structure
mp_e_above_hull	float	Energy above convex hull from MP database (eV/atom)
negative_freq_Gamma	boolean	Existence of negative frequencies at Γ-point
negative_freq_path	boolean	Existence of negative frequencies in phonon dispersion
Refs	string	Links to Phonon database and MP websites

### Database statistics

As shown in Fig. 2, there were 10032 materials in the Phonon database and 8382 of them were flagged for calculation. Since each structure contains several vibrational modes, the total number of modes in our database was 725163, and 428081 modes of them are Raman active or the activity is unknown (8533 modes).

**Fig. 3:**
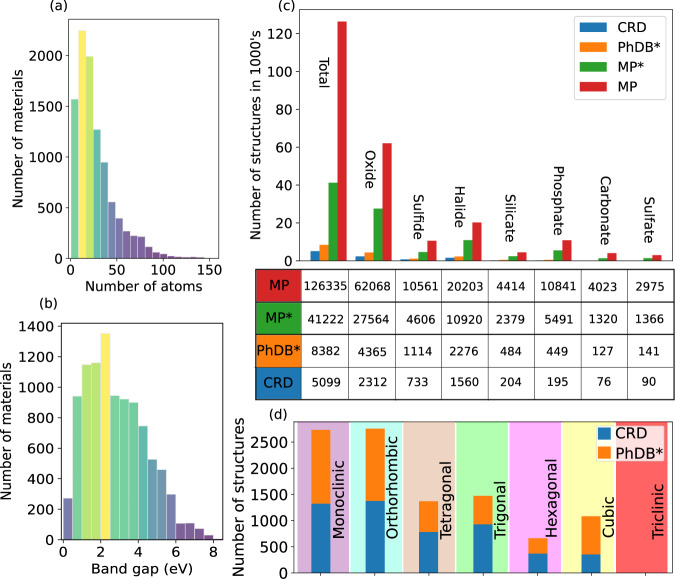
Database statistics. (**a,b**) The number of materials in Phonon database as a function of number of atoms in structures and band gap, respectively. (**c**) Comparison of the number of different types of compounds in Material Project (MP) and Computational Raman Database (CRD). MP* and PhDB* shows the number of structures in Materials Project and Phonon database, respectively, when the same selection conditions as in CRD are applied to them. (**d**) The number of materials in different space groups as grouped by the crystal system.

Figure 3a,b shows the number of materials in the database (before prescreening) grouped by the calculated band gaps and the number of atoms in their structures, respectively. The histogram with respect to the number of atoms, peaks at around 20–30. There are some materials with very large primitive cells containing more than 100 atoms, but many of these appear to be disordered/alloyed/defective variants of the small primitive cell systems and thus of limited interest. Since the Phonon database only includes non-metallic materials, the number of materials with a band gap smaller than 0.5 eV is small, and therefore neglecting those materials in our prescreening step has small impact.

We proceeded to carry out the Raman tensor calculations in the order of increasing number of atoms in the primitive cell. The database included here contains 5099 calculated structures. We calculated all materials with less than 10 atoms in the primitive cell and all experimentally observed materials (as indicated by MP) less than 40 atoms in the primitive cell. For this, we used about 9.5 million CPU hours. We estimate that for calculating the remaining 3283 structures would require more than 20 million CPU hours, owing to the much larger cell sizes.

In Fig. 3c we compare the number of materials considered in this work and in Materials Project database as grouped by the type of compound (oxides, halides, etc.). “MP” denotes the full Materials Project database, whereas “MP*“ includes the same conditions (band gap larger than 0.5 eV and energy above hull less than 0.1 eV) as used in our material set (PhDB*). “CRD” refers to the calculated set of materials. In total, almost 20% of the MP* structures are contained in the PhDB* dataset and about 12% are calculated. Also, the different types of compounds are included in our database with similar statistics as in Materials Project. As an example, the percentage of oxides and halogenides are 52% and 27% in our database, compared to 67% and 26% in MP*. Finally, we used the algorithm proposed by Larsen *et al*.^47^ for identifying the dimensionality of the structures in our database: 4137 structures (more than 80%) are three-dimensional, 385 structures are two-dimensional, 72 structures are one-dimensional, 277 structures are 0D and others are a mixture of different dimensionality, such as 0D + 1D, 0D + 2D, 0D + 3D, etc. Figure 3d shows the distribution of different space groups in our database (and in the Phonon database) as grouped by the crystal system. This shows that our database covers most different material classes.

## Technical Validation

### Comparison to experiments

Selected computational benchmarks were already presented in the Computational parameters section. In this section, we compare the calculated spectra from our approach with experimental results extracted from the RRUFF database to validate our method and calculations. RRUFF contains only (estimated) chemical formula and lattice parameters but not atomic positions, and thus we cannot guarantee exact structural match. Based on mineral names, there are 703 entries in RRUFF database that matched with 288 structures of our database. The Table S1 contains mineral names, formula, and their RRUFF IDs for structures with the same formula as found in Phonon database, 92 in total. 27 of these were found to have the similar lattice parameters compared to the matched structure in our database and thus very likely to be the same structure. Moreover, in most cases, the energy above hull is zero or very small, the maximum being 40 meV/atom.

**Fig. 4:**
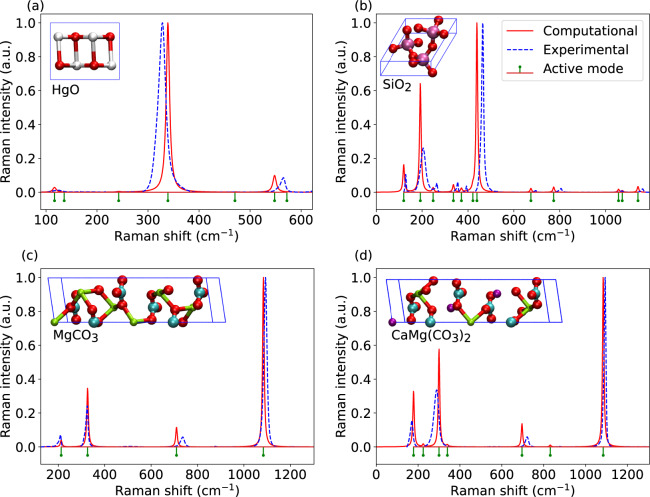
Comparison of calculated Raman spectra (red solid line) and experimental spectra from RRUFF database [10] (blue dashed line) for selected minerals. Green short line segments show the Raman active modes based on the symmetry analysis. Both spectra are normalized to one at maximum. The experimental spectra correspond to processed data measured at wavelength 532 nm from unoriented samples with RRUFF id: R140877, R050125, R050676, and R050129, for HgO, SiO_2_, MgCO_3_, and CaMg(CO_3_)_2_, respectively. The atomic structures are given in the inset (O: red, Hg: white, Si: magenta, Mg: green, C: cyan, Ca: purple).

Figure 4 shows a comparison between calculated spectra and experimental Raman spectra of few selected minerals: HgO, MgCO_3_, CaMg(CO_3_)_2_, and SiO_2_. Overall good agreement between computational and experimental results is found. We note that the comparison to the experiment is complicated by the varying linewidths in the experimental spectra, which in turn modifies the peak maxima. The linewidth is related to the phonon lifetime, which is not evaluated in our calculations. Instead, in the simulated spectra we have only included a reasonable phonon lifetime-induced broadening of 8 cm^−1^ to allow for more straightforward visual comparison.

While perfectly ordered bulk crystals are used in calculations, in experiments the material purity or even exact composition may be unknown and the spectrum is affected by parameters such as temperature, pressure, and measurement geometry. While we are relying in harmonic approximation, phonon renormalization due to anharmonic effects can affect the frequencies as well as linewidths. Also, we are simulating non-resonant Raman spectra, while in resonant Raman the intensities may change depending on the electronic resonance conditions. Nevertheless, in the cases where the Raman tensors are affected by any of these effects, the Raman-active modes found based on the group theory can still be used to assist in the analysis of the experimental spectra.

**Fig. 5:**
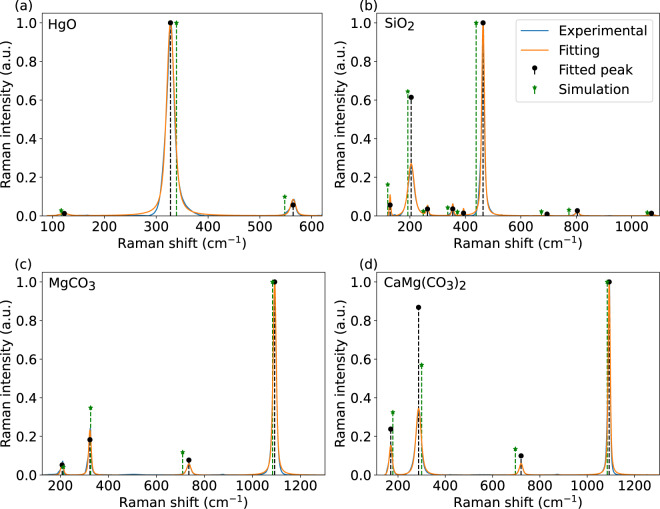
Comparison of experimental Raman spectra (blue solid line) from RRUFF database and fitted spectra (orange solid line) for selected minerals. Green dashed line with star and black dashed line with circle show calculated Raman peaks and fitted peaks, respectively. All values are normalized to one at maximum. The experimental spectra correspond to processed data measured at wavelength 532 nm from unoriented samples with RRUFF id: R140877, R050125, R050676, and R050129, for HgO, SiO_2_, MgCO_3_, and CaMg(CO_3_)_2_, respectively.

For a more quantitative comparison to experiments, we carried out peak fitting to the experimental spectra using a Voigt lineshape. Figure 5 shows the fitted spectra with respect to the experimental Raman spectra and a comparison between simulated and fitted peak positions and intensities. Average frequency and intensity differences and their standard deviations are listed in Table S3. As all calculated frequencies of SiO_2_ are lower than in experiments, it exhibits the largest average difference of 18.5 cm^−1^, but the smallest deviation of 6 cm^−1^. In the case of CaMg(CO_3_)_2_, some calculated frequencies are overestimated and some underestimated, and thus it shows the smallest average difference of 2.6 cm^−1^, but the largest deviation of 14.8 cm^−1^. In the case of intensities, normalization makes the comparison less straightforward, but the standard deviation is found to vary between 0.02–0.15.

## Usage Notes

We introduced an optimized workflow for performing high-throughput first-principles calculations of Raman tensors. The workflow takes full advantage of the crystal symmetry, adopts carefully benchmarked computational parameters, and avoids calculation of vibrational modes by importing them from existing Phonon database. We carried out such calculations for 5099 materials and the results are included in the dataset accompanying this paper. The database encompasses a wide variety of materials from different compound classes (oxides, halides, etc.) and of different dimensionality. The calculated spectra were also shown to compare favorably with the experimental ones.

The final database contains Raman tensors and other vibrational information, such as phonon eigenmodes, Born charges (adopted from Phonon database), and symmetry information, stored in JSON document that can be downloaded directly from the Materials Cloud Archive^46^ and queried with a simple python script. The whole dataset can also be browsed online in Computational Raman Database website (https://ramandb.oulu.fi), wherein one can also find other relevant information, such as atomic structure, phonon dispersion, and infrared spectrum. We hope that the vibrational properties and Raman spectra of materials in the database will prove useful for computational and experimental researchers alike.

## Supplementary information

Supplementary Information

## Data Availability

VASP^34,35^ used in all DFT calculations is a proprietary software. For the database, dimensionality analysis, and web app, we used Atomic Simulation Environment (ASE) and Atomic Simulation Recipes (ASR)^36,37^, both released under GNU Lesser General Public License (LGPL). Phonopy^31^ used in calculating the eigenvectors and performing symmetry analysis is released under New Berkeley Software Distribution (BSD) License. The workflow is defined as a part of Atomate code package^38^ with FireWorks^39^ for defining, managing, and executing jobs which both are released under a modified BSD license and free to the public. Pymatgen (Python Materials Genomics) used for producing inputs parameters and custodian^40^ for performing error checking are both open-source packages under Massachusetts Institute of Technology (MIT) license. To store results and task parameters, MongoDB NoSQL database was used with the Server Side Public License (SSPL). All the information for prescreening and phonon calculation extracted from Phonon Database^17,31^ and from Materials project^32,48^ are both released under Creative Commons Attribution 4.0 International License. Fitting analysis of the experimental spectra was performed by Least-Squares Minimization fitting (LMfit)^49^ python package released under New Berkeley Software Distribution (BSD) license.
