# Supplementary material for: High-throughput computation of Raman spectra from first principles
Source: Sci Data. 2023 Feb 8;10:80. doi: 10.1038/s41597-023-01988-5 (PMC9908888; doi:10.1038/s41597-023-01988-5)
Supplement: Supplementary file 1 — Supplementary Information [file 41597_2023_1988_MOESM1_ESM.pdf]

# Supplemental Material to "High-throughput computation of Raman spectra from first principles"

**Mohammad Bagheri**[1] **and Hannu-Pekka Komsa**[1,*]

[1]Microelectronics Research Unit, Faculty of Information Technology and Electrical Engineering, University of Oulu, Oulu, FIN-90014, Finland
[*]corresponding author(s): Hannu-Pekka Komsa (hannu-pekka.komsa@oulu.fi)

## Contents

Benchmark 1
References 10

## List of Tables

S1 Common structures in our database based on the same chemical formula and the mineral name in RRUFF compared to Materials Project tags. The bold RRUFF IDs refer to structures that have also similar lattice parameters. . . . . 4
S2 Raman activity of all point groups and irreducible representation (irrep.) based on group theory. True means active and False means inactive . . . . . 6
S3 Comparison of calculated frequencies and intensities with those obtained from the fits to the experimental spectra shown in Figure 5. Frequency and intensity differences are defined as $\Delta\omega = \omega_{\text{fit}} - \omega_{\text{calc}}$ and $\Delta I = I_{\text{fit}} - I_{\text{calc}}$, where $I$ is the normalized intensity. Std refers to standard deviation. . . . . 6

## List of Figures

S1 The effect of k-point mesh parameter $R_k$. (a) and (b) show the unnormalized Raman activity of AlN and Si, respectively, with $R_k$ = 20 (blue line), 30 (orange line), 40 (green line) and 60 (red line). (c) and (d) show the effects of different $R_k$ on the maximum intensity of AlN and Si spectra, respectively. (e) and (f) show the effects of different $R_k$ on the average dielectric constant of AlN and Si, respectively. . . 7
S2 Changes of dielectric constant of (a) PbO and (b) $Cd(HO)_2$ in different directions as a function of the displacement step size (0.005–0.04). . . . . 8
S3 Comparison of the Raman activity from our workflow and that from vasp_raman code. The spectra from old version of Atomate with incorrect eigenvector normalization is also shown. . . . . 9

## Benchmark

To benchmark of our approach, we selected four materials with different band gaps and different atomic masses: Si, PbO, AlN, and $Cd(HO)_2$. We first verified that the standard approach of calculating Raman-tensors for all modes agrees with the Raman-active modes identified using group theory. That is, all the Raman-inactive modes were found to have vanishingly small Raman tensor, although often nonzero due to numerical errors.

Next, we investigated the effect of k-point mesh density on Raman tensors and Raman spectra. We calculated Raman tensors for $R_k$ = 30, 40 and 60 and compared the results with the standard value ($R_k$ = 20) used in Phonon database. Results from two (out of four) materials are shown in Fig. S1, where, to represent large and small bandgap materials, we selected AlN (4.05 eV) and Si (0.85 eV), respectively. Fig. S1(a,b) shows the unnormalized Raman activity spectra for different $R_k$, which clearly illustrates that AlN is hardly affected whereas Si experiences significant changes, thus suggesting that $R_k$ should be increased. To better illustrate the magnitude of changes, Fig. S1(c,d) show how the maximum intensity changes with increasing $R_k$ and Fig. S1(e,f) shows the average dielectric constant. In the case of AlN, $R_k$ = 20 already yields Raman tensors within 10 % of the converged value and dielectric constant within 1 %. In the case of Si, $R_k$ = 40 is required to reach similar accuracy. Based on these results we

decided to use $R_k$=40 for materials with bandgap smaller than 1 eV, $R_k$=30 for materials with bandgap between 1 eV to 2 eV, and $R_k$=20 for materials with a bandgap greater than 2 eV.

In the third step, we investigated the effect of step size by calculating Raman tensors of PbO and $Cd(HO)_2$ with step sizes of 0.001, 0.02, and 0.04 Å and compared them with those using the standard step size of 0.005 Å. Fig. S2 shows the changes in dielectric constants of PbO and $Cd(HO)_2$ in different directions and plotted for three-step sizes: 0.005, 0.02, and 0.04 Å. Since dielectric tensor is symmetric (xy=yx, xz=zx, and yz=zy), we only plot the inequivalent components. As shown in Fig. S2, whenever there are pronounced changes in the dielectric constant (corresponding to non-zero components in Raman tensor), the dependence on step size is close to linear. In some cases there is a small parabolic dependence, seen particularly well in the xy=yx component which contains no linear dependence, but these will not affect the Raman tensor since we are using two-point finite-difference stencil. Moreover, in this range of step sizes there is no discernible noise, although some noise could be observed in 0.001 Å results (not shown). Thus, we consider the default value of 0.005 Å a good choice.

As mentioned in the text, there was an error in the normalization of eigenvectors in Atomate. We fixed the normalization error and changed the formulations to match with the vasp_raman code[1,2]. To verify our approach, we used vasp_raman code to calculate Raman tensors and compared them to Atomate with the fixed and old versions of eigenvector normalization. Fig. S3 shows the Raman activity spectra of $MoS_2$, $WS_2$, SrGaSnH, and BaAlSiH. The revised normalization yields activities closely matching with vasp_raman code. The incorrect normalization, on the other hand, tends to lead to overestimation of Raman activities and is particularly severe with modes that have very small Raman activity.

In CRD website (https://ramandb.oulu.fi), the total Raman intensity is separated into depolarized ($I_\perp$) and polarized ($I_{||}$) components, $I = I_\perp + I_{||}$, with

$$\frac{I_{||}}{(\omega_L-\omega_\nu)^4} \sim \frac{\hbar(n+1)}{30\omega_\nu}(10G_\nu^{(0)}+4G_\nu^{(2)}) \tag{1}$$

$$\frac{I_{\perp}}{(\omega_L-\omega_\nu)^4} \sim \frac{\hbar(n+1)}{30\omega_\nu}(5G_\nu^{(1)}+3G_\nu^{(2)}) \tag{2}$$

where we have taken out the $(\omega_L-\omega_\nu)^4$ term that depends on the laser wavelength, since (i) this removes the dependence on one external parameter from our spectra, (ii) our calculations are for non-resonant conditions and one needs to be careful to only compare to wavelengths that are far from resonance, and (iii) the dependence on $\omega_\nu$ and thus the changes in the spectra after normalization are usually small. The rotation invariants are[3,4]

$$G_\nu^{(0)} = \frac{1}{3}(R_{\nu xx}+R_{\nu yy}+R_{\nu zz})^2 \tag{3}$$

$$G_\nu^{(1)} = \frac{1}{2}[(R_{\nu xy}-R_{\nu yx})^2+(R_{\nu xz}-R_{\nu zx})^2+(R_{\nu zy}-R_{\nu yz})^2] \tag{4}$$

$$G_\nu^{(2)} = \frac{1}{2}[(R_{\nu xy}+R_{\nu yx})^2+(R_{\nu xz}+R_{\nu zx})^2+(R_{\nu zy}+R_{\nu yz})^2] + \frac{1}{3}[(R_{\nu xx}-R_{\nu yy})^2+(R_{\nu xx}-R_{\nu zz})^2+(R_{\nu zz}-R_{\nu yy})^2] \tag{5}$$

| Mineral name | Formula | mpid | Energy above hull (eV) | RRUFF ID |
|---|---|---|---|---|
| Billingsleyite | $Ag_7AsS_6$ | mp-15077 | 0.003 | **R070350** |
| Sanbornite | $BaSi_2O_5$ | mp-3031 | 0 | **R060489** |
| Hardystonite | $Ca_2ZnSi_2O_7$ | mp-6227 | 0.015 | **R040026** |
| Perovskite | $CaTiO_3$ | mp-4019 | 0 | **R050456** |
| Greenockite | CdS | mp-672 | 0 | **R090045** |
| Cobaltite | CoAsS | mp-4627 | 0.001 | **R070372** |
| Cobaltite | CoAsS | mp-16363 | 0.004 | **R060907** |
| Cuprite | $Cu_2O$ | mp-361 | 0 | **R050374** |
| Stromeyerite | CuAgS | mp-5014 | 0.024 | **R060908** |
| Emplectite | $CuBiS_2$ | mp-22982 | 0 | **R070307** |

Continued on next page

**Table S1 – continued from previous page**

| Mineral name | Formula | mpid | Energy above hull (eV) | RRUFF ID |
|---|---|---|---|---|
| Chalcostibite | $CuSbS_2$ | mp-4468 | 0 | **R060262** |
| Pyrite | $FeS_2$ | mp-226 | 0.008 | **R050070** |
| Marcasite | $FeS_2$ | mp-1522 | 0 | **R060882** |
| Langbeinite | $K_2Mg_2(SO_4)_3$ | mp-6299 | 0 | **R070285** |
| Aphthitalite | $K_3Na(SO_4)_2$ | mp-22457 | 0 | **R050651** |
| Goldschmidtite | $KNbO_3$ | mp-7375 | 0 | **R190009** |
| Nordite-(La) | $Na_3SrLaZnSi_6O_{17}$ | mp-13726 | 0 | **R140310** |
| Swedenborgite | $NaBe_4SbO_7$ | mp-8075 | 0 | **R060486** |
| Leucophanite | $NaCaBeSi_2O_6F$ | mp-560721 | 0 | **R050004** |
| Neighborite | $NaMgF_3$ | mp-2955 | 0 | **R080108** |
| Cotunnite | $PbCl_2$ | mp-23291 | 0.006 | **R060655** |
| Matlockite | PbClF | mp-22964 | 0 | **R140538** |
| Laurite | $RuS_2$ | mp-2030 | 0 | **R110120** |
| Zincite | ZnO | mp-2133 | 0 | **R060027** |
| Chrysoberyl | $BeAl_2O_4$ | mp-3081 | 0 | **R040073** |
| Wurtzite | ZnS | mp-10281 | 0.002 | **R130069** |
| Montroydite | HgO | mp-1224 | 0 | **R070235** |
| Quartz | $SiO_2$ | mp-7000 | 0.011 | **R050125** |
| Bromellite | BeO | mp-2542 | 0 | X050194 |
| Litharge | PbO | mp-19921 | 0.001 | R060959 |
| Romarchite | SnO | mp-2097 | 0 | R080006 |
| Anatase | $TiO_2$ | mp-390 | 0.006 | R060277 |
| Andalusite | $Al_2SiO_5$ | mp-4753 | 0 | R050258 |
| Anglesite | $Pb(SO_4)$ | mp-3472 | 0 | R040004 |
| Aragonite | $CaCO_3$ | mp-4626 | 0.024 | R040078 |
| Baryte | $Ba(SO_4)$ | mp-3164 | 0 | R040036 |
| Brenkite | $Ca_2CO_3F_2$ | mp-6246 | 0.028 | R060247 |
| Calcite | $CaCO_3$ | mp-3953 | 0 | R040070 |
| Cerussite | $Pb(CO_3)$ | mp-19893 | 0 | R040069 |
| Colquiriite | $CaLiAlF_6$ | mp-1224 | 0 | R070417 |
| Dolomite | $CaMg(CO_3)_2$ | mp-6459 | 0 | R050129 |
| Eitelite | $Na2Mg(CO_3)_2$ | mp-6026 | 0 | R110214 |
| Eulytine | $Bi_4(SiO_4)_3$ | mp-23331 | 0 | R060058 |
| Farringtonite | $Mg_3(PO_4)_2$ | mp-14396 | 0 | R130127 |
| Geikielite | $MgTiO_3$ | mp-3771 | 0 | R070479 |
| Glauberite | $Na_2Ca(SO_4)_2$ | mp-6397 | 0 | R050350 |
| Huntite | $CaMg_3(CO_3)_4$ | mp-6524 | 0.004 | R040126 |
| Cristobalite | $SiO_2$ | mp-6945 | 0.003 | R070235 |
| Leiteite | $ZnAs_2O_4$ | mp-29509 | 0.006 | R040011 |
| Lithiophosphate | $Li_3(PO_4)$ | mp-2878 | 0.001 | R100092 |
| Magnesite | $Mg(CO_3)$ | mp-5348 | 0 | R040114 |
| Nahcolite | $NaH(CO_3)$ | mp-696396 | 0 | R070237 |
| Witherite | $Ba(CO_3)$ | mp-5504 | 0 | R040040 |
| Arsenolite | $As_2O_3$ | mp-2184 | 0.009 | R050383 |
| Åkermanite | $Ca_2MgSi_2O_7$ | mp-6094 | 0.023 | R061085 |
| Benitoite | $BaTi(SiO_3)_3$ | mp-6661 | 0 | R050320 |
| Gahnite | $ZnAl_2O_4$ | mp-2908 | 0 | R070591 |
| Rosiaite | $PbSb_2O_6$ | mp-20727 | 0 | R070384 |
| Xanthoconite | $Ag_3AsS_3$ | mp-561620 | 0 | R070746 |
| Topaz | $Al_2SiO_4F_2$ | mp-6280 | 0 | R040121 |

Continued on next page

**Table S1 – continued from previous page**

| Mineral name | Formula | mpid | Energy above hull (eV) | RRUFF ID |
|---|---|---|---|---|
| Imiterite | $Ag_2HgS_2$ | mp-9635 | 0.03 | R080014 |
| Acanthite | $Ag_2S$ | mp-610517 | 0.024 | R070578 |
| Argyrodite | $Ag_8GeS_6$ | mp-9770 | 0 | R050437 |
| Andalusite | $Al_2SiO_5$ | mp-4934 | 0.007 | R050258 |
| Nitrobarite | $Ba(NO_3)_2$ | mp-4396 | 0 | R060622 |
| Barylite | $BaBe_2Si_2O_7$ | mp-6383 | 0 | R060620 |
| Barylite | $BaBe_2Si_2O_7$ | mp-12797 | 0 | R060606 |
| Guanajuatite | $Bi_2Se_3$ | mp-23164 | 0.028 | R080140 |
| Merwinite | $Ca_3Mg(SiO_4)_2$ | mp-558209 | 0.038 | R070195 |
| Rankinite | $Ca_3Si_2O_7$ | mp-3932 | 0.009 | R140775 |
| Hurlbutite | $CaBe_2(PO_4)_2$ | mp-6772 | 0 | R090048 |
| Rynersonite | $CaTa_2O_6$ | mp-18229 | 0 | R080064 |
| Arsenopyrite | FeAsS | mp-561511 | 0 | R050071 |
| Gudmundite | FeSbS | mp-27904 | 0 | R060741 |
| Cinnabar | HgS | mp-634 | 0.004 | R070532 |
| Cinnabar | HgS | mp-9252 | 0.004 | R070532 |
| Kalsilite | $KAlSiO_4$ | mp-8355 | 0.002 | R060801 |
| Kalsilite | $KAlSiO_4$ | mp-9480 | 0.002 | R060030 |
| Avogadrite | $KBF_4$ | mp-4929 | 0 | R110062 |
| Kotoite | $Mg_3(BO_3)_2$ | mp-5005 | 0 | R060940 |
| Natrosilite | $Na_2Si_2O_5$ | mp-3193 | 0 | R060855 |
| Molybdomenite | $PbSeO_3$ | mp-20716 | 0 | R140388 |
| Valentinite | $Sb_2O_3$ | mp-2136 | 0 | R120096 |
| Stibnite | $Sb_2S_3$ | mp-2809 | 0 | R120137 |
| Moissanite | SiC | mp-7631 | 0 | R150016 |
| Tellurite | $TeO_2$ | mp-2125 | 0 | R070606 |
| Rutile | $TiO_2$ | mp-2657 | 0.037 | R060745, R120008 |
| Brookite | $TiO_2$ | mp-1840 | 0.02 | R050363, R050591, R130225 |
| Lorándite | $TlAsS_2$ | mp-4988 | 0 | R110055 |
| Tungstenite | $WS_2$ | mp-224 | 0 | R070616 |
| Waimirite-(Y) | $YF_3$ | mp-2416 | 0 | R130714 |
| Reinerite | $Zn_3(AsO_3)_2$ | mp-27580 | 0 | R080132 |
| Baddeleyite | $ZrO_2$ | mp-2858 | 0 | R100171 |

**Table S1.** Common structures in our database based on the same chemical formula and the mineral name in RRUFF compared to Materials Project tags. The bold RRUFF IDs refer to structures that have also similar lattice parameters.

| Point group | Irrep. label | Raman Activity | Point group | Irrep. label | Raman Activity |
|---|---|---|---|---|---|
| $C_1$(1) | A | True | $C_6$ (6) | A | True |
| $C_i$(-1) | $A_g$ | True | | B | False |
| | $A_u$ | False | | $E_1$ | True |
| $C_2$ (2) | A | True | | $E_2$ | True |
| | B | True | $C_{3h}$ (-6) | A′ | True |
| $C_{1v} = C_s = C_{1h}$ (m) | A′ | True | | E′ | True |
| | A″ | True | | A″ | False |
| $C_{2h}$ (2/m) | $A_g$ | True | | E″ | True |
| | $B_g$ | True | $C_{6h}$ (6/m) | $A_g$ | True |
| | $A_u$ | False | | $B_g$ | False |
| | $B_u$ | False | | $E_{1g}$ | True |

Continued on next page

**Table S2 – continued from previous page**

| Point group | Irrep. label | Raman Activity | Point group | Irrep. label | Raman Activity |
|---|---|---|---|---|---|
| $D_2$ (222) | A | True | | $E_{2g}$ | True |
| | $B_1$ | True | | $A_u$ | False |
| | $B_2$ | True | | $B_u$ | False |
| | $B_3$ | True | | $E_{1u}$ | False |
| $C_{2v}$ (mm2) | $A_1$ | True | | $E_{2u}$ | False |
| | $A_2$ | True | $D_6$ (622) | $A_1$ | True |
| | $B_1$ | True | | $A_2$ | False |
| | $B_2$ | True | | $B_1$ | False |
| $D_{2h}$ (mmm) | $A_g$ | True | | $B_2$ | False |
| | $B_{1g}$ | True | | $E_1$ | True |
| | $B_{2g}$ | True | | $E_2$ | True |
| | $B_{3g}$ | True | $C_{6v}$ (6mm) | $A_1$ | True |
| | $A_u$ | False | | $A_2$ | False |
| | $B_{1u}$ | False | | $B_1$ | False |
| | $B_{2u}$ | False | | $B_2$ | False |
| | $B_{3u}$ | False | | $E_1$ | True |
| $C_4$ (4) | A | True | | $E_2$ | True |
| | B | True | $D_{3h}$ (-6m2) | $A'_1$ | True |
| | E | True | | $A'_2$ | False |
| $S_4$ (-4) | A | True | | E′ | True |
| | B | True | | $A''_1$ | False |
| | E | True | | $A''_2$ | False |
| $C_{4h}$ (4/m) | $A_g$ | True | | E″ | True |
| | $B_g$ | True | $D_{6h}$ (6/mmm) | $A_{1g}$ | True |
| | $E_g$ | True | | $A_2g$ | False |
| | $A_u$ | False | | $B_{1g}$ | False |
| | $B_u$ | False | | $B_{2g}$ | False |
| | $E_u$ | False | | $E_{1g}$ | True |
| $D_4$ (422) | $A_1$ | True | | $E_{2g}$ | True |
| | $A_2$ | False | | $A_{1u}$ | False |
| | $B_1$ | True | | $A_{2u}$ | False |
| | $B_2$ | True | | $B_{1u}$ | False |
| | E | True | | $B_{2u}$ | False |
| $C_{4v}$ (4mm) | $A_1$ | True | | $E_{1u}$ | False |
| | $A_2$ | False | | $E_{2u}$ | False |
| | $B_1$ | True | T (23) | A | True |
| | $B_2$ | True | | E | True |
| | E | True | | T | True |
| $D_{2d}$ (-42m) | $A_1$ | True | $T_h$ (m-3) | $A_g$ | True |
| | $A_2$ | False | | $A_u$ | False |
| | $B_1$ | True | | $E_g$ | True |
| | $B_2$ | True | | $E_u$ | False |
| | E | True | | $T_g$ | True |
| $D_{4h}$ (4/mmm) | $A_{1g}$ | True | | $T_u$ | False |
| | $A_{2g}$ | False | O (432) | $A_1$ | True |
| | $B_{1g}$ | True | | $A_2$ | False |
| | $B_{2g}$ | True | | E | True |
| | $E_g$ | True | | $T_1$ | False |
| | $A_{1u}$ | False | | $T_2$ | True |
| | $A_{2u}$ | False | $T_d$ (-43m) | $A_1$ | True |
| | $B_{1u}$ | False | | $A_2$ | False |
| | $B_{2u}$ | False | | E | True |

Continued on next page

**Table S2 – continued from previous page**

| Point group | Irrep. label | Raman Activity | Point group | Irrep. label | Raman Activity |
|---|---|---|---|---|---|
| | $E_u$ | False | | $T_1$ | False |
| $C_3$ (3) | A | True | | $T_2$ | True |
| | E | True | $O_h$ (m-3m) | $A_{1g}$ | True |
| $S_6$ (-3) | $A_g$ | True | | $A_{2g}$ | False |
| | $E_g$ | True | | $E_g$ | True |
| | $A_u$ | False | | $T_{1g}$ | False |
| | $E_u$ | False | | $T_{2g}$ | True |
| $D_3$ (32) | $A_1$ | True | | $A_{1u}$ | False |
| | $A_2$ | False | | $A_{2u}$ | False |
| | E | True | | $E_u$ | False |
| $C_{3v}$ (3m) | $A_1$ | True | | $T_{1u}$ | False |
| | $A_2$ | False | | $T_{2u}$ | False |
| | E | True | | | |
| $D_{3d}$ (-3m) | $A_{1g}$ | True | | | |
| | $A_{2g}$ | False | | | |
| | $E_g$ | True | | | |
| | $A_{1u}$ | False | | | |
| | $A_{2u}$ | False | | | |
| | $E_u$ | False | | | |

**Table S2.** Raman activity of all point groups and irreducible representation (irrep.) based on group theory. True means active and False means inactive

| Formula | Avg[$\Delta\omega$] ($cm^{-1}$) | Avg[$\Delta I$] (a.u.) | Std(Freq) ($cm^{-1}$) | Std(I) (a.u.) |
|---|---|---|---|---|
| HgO | 3.52 | -0.02 | 11.35 | 0.02 |
| $SiO_2$ | 18.54 | -0.02 | 5.95 | 0.03 |
| $MgCO_3$ | 6.67 | -0.05 | 12.26 | 0.07 |
| $CaMg(CO_3)_2$ | 2.61 | 0.04 | 14.78 | 0.15 |

**Table S3.** Comparison of calculated frequencies and intensities with those obtained from the fits to the experimental spectra shown in Figure 5. Frequency and intensity differences are defined as $\Delta\omega = \omega_{\text{fit}} - \omega_{\text{calc}}$ and $\Delta I = I_{\text{fit}} - I_{\text{calc}}$, where $I$ is the normalized intensity. Std refers to standard deviation.

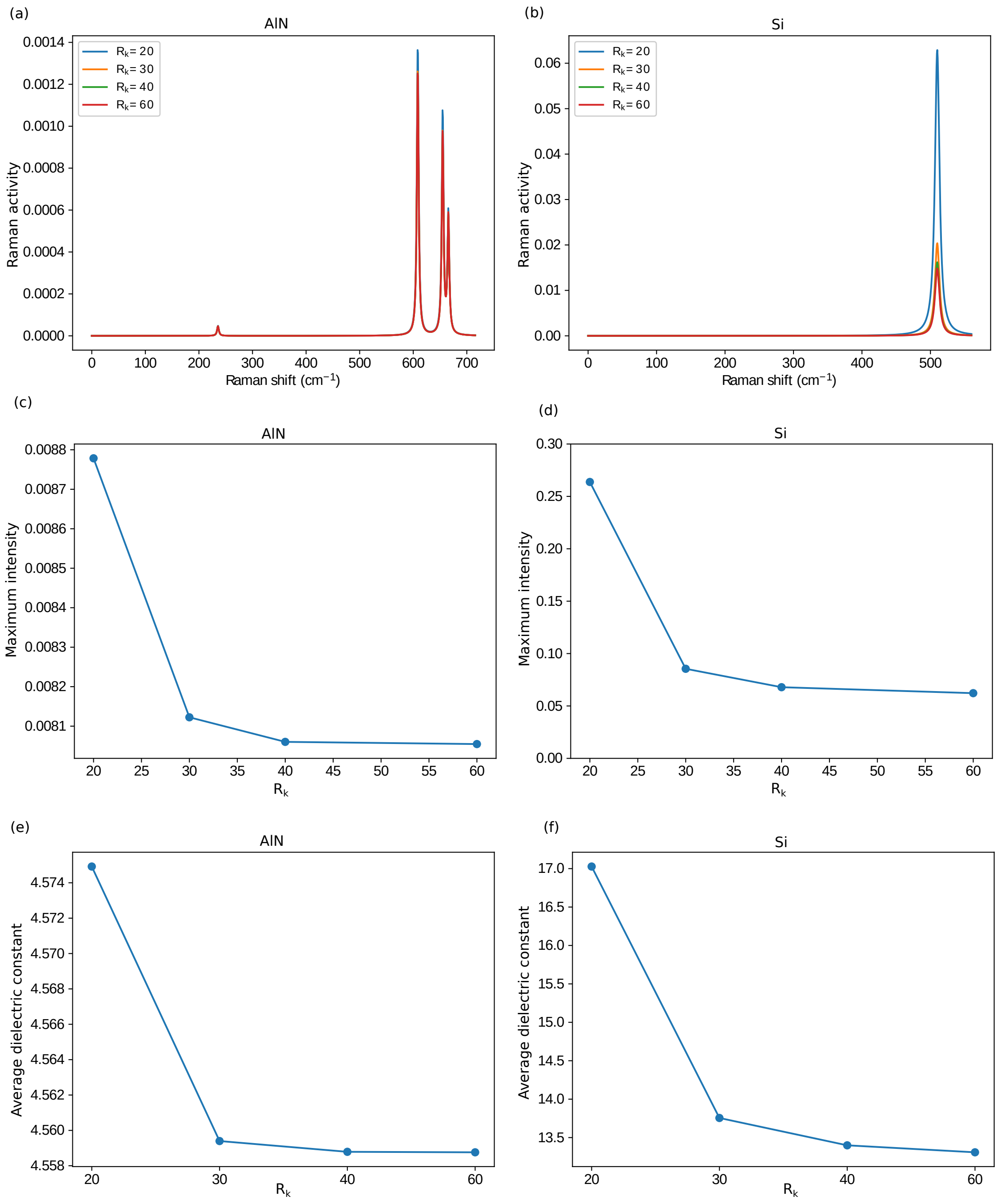


**Figure S1.** The effect of k-point mesh parameter $R_k$. (a) and (b) show the unnormalized Raman activity of AlN and Si, respectively, with $R_k$ = 20 (blue line), 30 (orange line), 40 (green line) and 60 (red line). (c) and (d) show the effects of different $R_k$ on the maximum intensity of AlN and Si spectra, respectively. (e) and (f) show the effects of different $R_k$ on the average dielectric constant of AlN and Si, respectively.

(a)

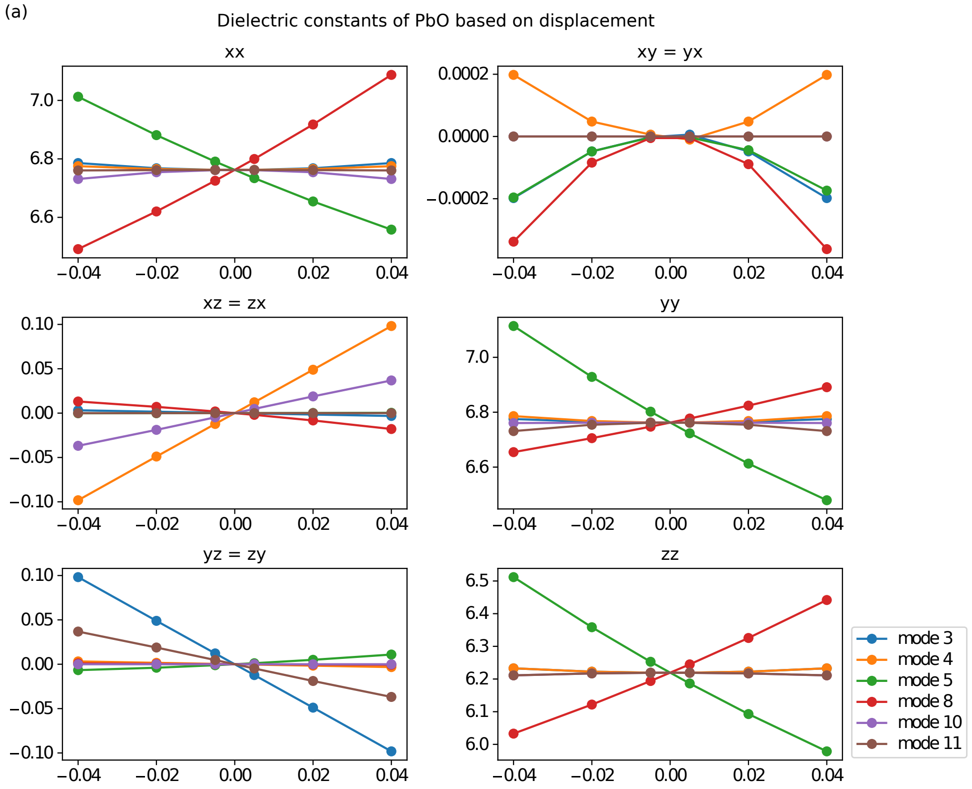


(b)

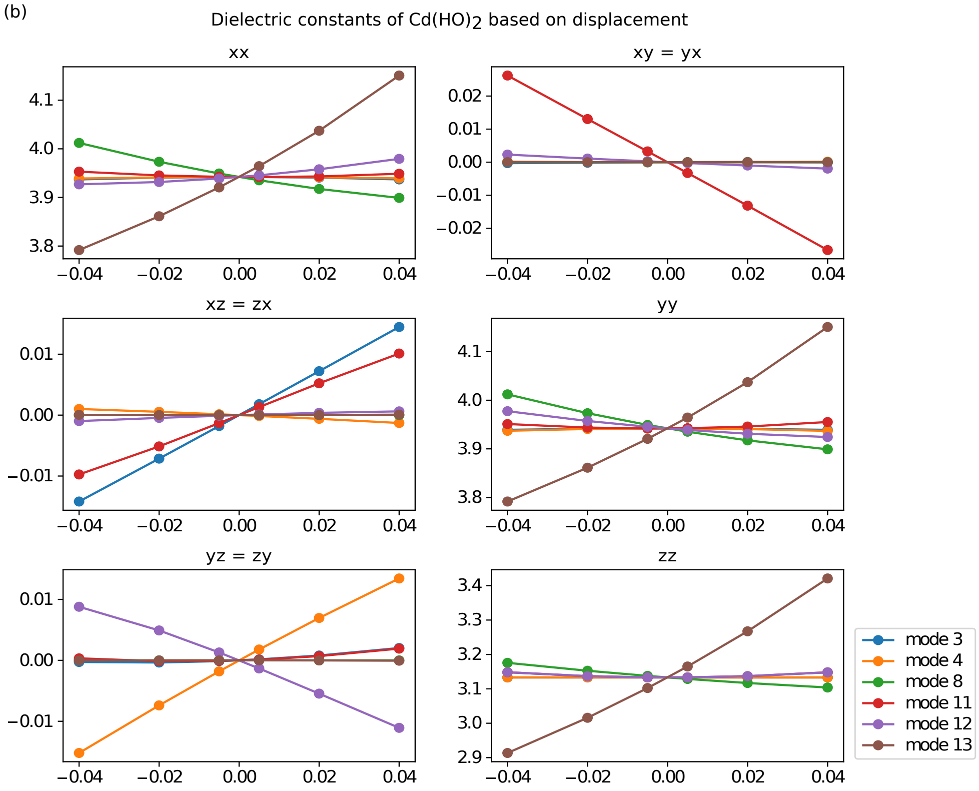


**Figure S2.** Changes of dielectric constant of (a) PbO and (b) $Cd(HO)_2$ in different directions as a function of the displacement step size (0.005–0.04).

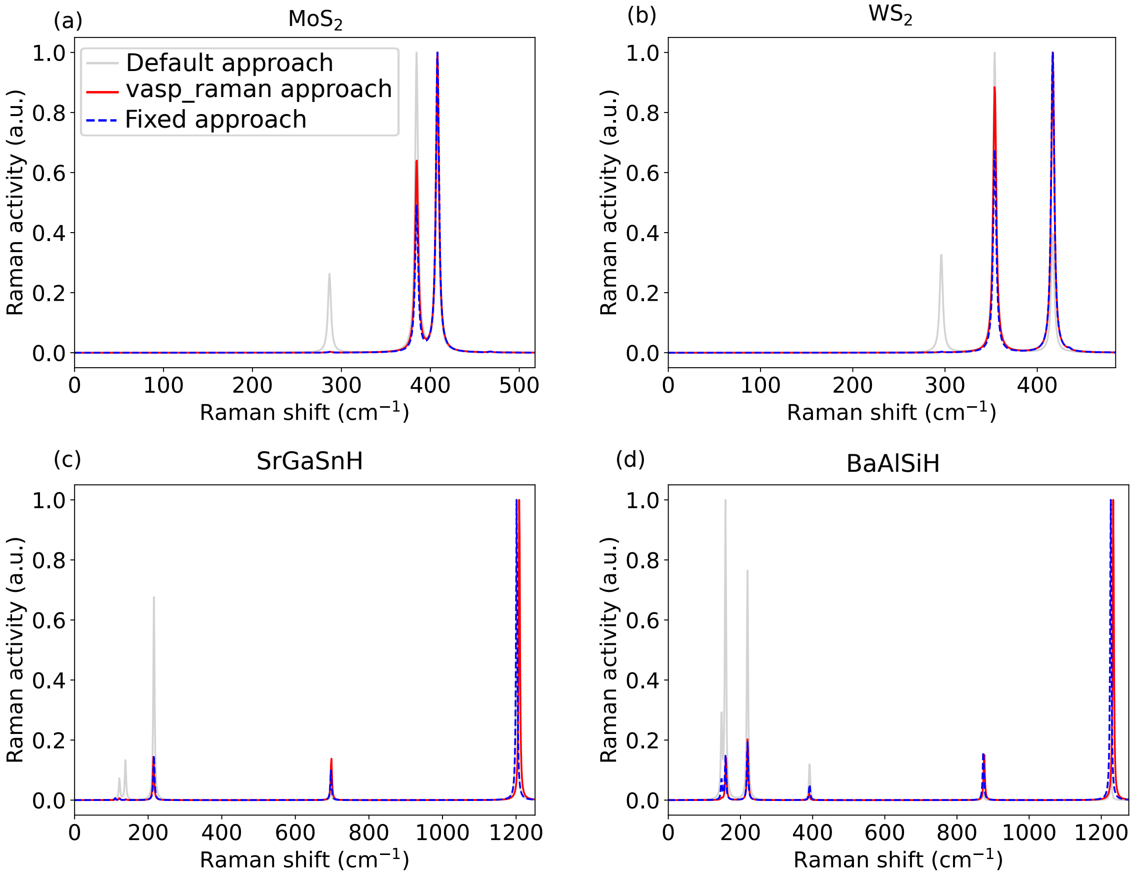


**Figure S3.** Comparison of the Raman activity from our workflow and that from vasp_raman code. The spectra from old version of Atomate with incorrect eigenvector normalization is also shown.

## References


1. Fonari, A. & Stauffer, S. Source code for: Python program to evaluate off-resonance raman activity using vasp code as the backend. *Github* https://github.com/raman-sc/VASP/ (2013).

2. Porezag, D. & Pederson, M. R. Infrared intensities and Raman-scattering activities within density-functional theory. *Phys. Rev. B - Condens. Matter Mater. Phys.* **54**, 7830–7836, https://doi.org/10.1103/PhysRevB.54.7830 (1996).

3. Prosandeev, S. A., Waghmare, U., Levin, I. & Maslar, J. First-order Raman spectra of $AB'_{1/2}B''_{1/2}O_3$ double perovskites. *Phys. Rev. B - Condens. Matter Mater. Phys.* **71**, 1–9, https://doi.org/10.1103/PhysRevB.71.214307 (2005).

4. Long, D. A. *The Raman effect* (John Wiley & Sons, Chichester, England, 2002).
